# Neuropathology of RAN translation proteins in fragile X-associated tremor/ataxia syndrome

**DOI:** 10.1186/s40478-019-0782-7

**Published:** 2019-10-30

**Authors:** Amy Krans, Geena Skariah, Yuan Zhang, Bryana Bayly, Peter K. Todd

**Affiliations:** 1 University of Michigan Medical School, Ann Arbor, USA; 2 Ann Arbor VA Medical Center, Ann Arbor, MI USA

**Keywords:** FXTAS, Protein aggregation, RAN translation, Neurodegeneration

## Abstract

CGG repeat expansions in *FMR1* cause the neurodegenerative disorder Fragile X-associated Tremor/Ataxia Syndrome (FXTAS). Ubiquitinated neuronal intranuclear inclusions (NIIs) are the neuropathological hallmark of FXTAS. Both sense strand derived CGG repeats and antisense strand derived CCG repeats support non-AUG initiated (RAN) translation of homopolymeric proteins in potentially 6 different reading frames. However, the relative abundance of these proteins in FXTAS brains and their co-localization with each other and NIIs is lacking. Here we describe rater-blinded assessment of immunohistochemical and immunofluorescence staining with newly generated antibodies to different CGG RAN translation products in FXTAS and control brains as well as co-staining with ubiquitin, p62/SQSTM1, and ubiquilin 2. We find that both FMRpolyG and a second CGG repeat derived RAN translation product, FMRpolyA, accumulate in aggregates in FXTAS brains. FMRpolyG is a near-obligate component of both ubiquitin-positive and p62-positive NIIs in FXTAS, with occurrence of aggregates in 20% of all hippocampal neurons and > 90% of all inclusions. A subset of these inclusions also stain positive for the ALS/FTD associated protein ubiquilin 2. Ubiquitinated inclusions and FMRpolyG+ aggregates are rarer in cortex and cerebellum. Intriguingly, FMRpolyG staining is also visible in control neuronal nuclei. In contrast to FMRpolyG, staining for FMRpolyA and CCG antisense derived RAN translation products were less abundant and less frequent components of ubiquitinated inclusions. In conclusion, RAN translated FMRpolyG is a common component of ubiquitin and p62 positive inclusions in FXTAS patient brains.

**Electronic supplementary material:**

The online version of this article (10.1186/s40478-019-0782-7) contains supplementary material, which is available to authorized users.

## Introduction

Nucleotide repeat expansions cause more than 40 neurological diseases, including Huntington disease, C9ORF72-associated Amyotrophic Lateral Sclerosis and Frontotemporal Dementia, and a number of spinocerebellar ataxias and myotonic dystrophies [1]. Repeat expansions cause disease through a series of overlapping mechanisms, including loss of expression of the repeat containing gene, gain of function toxicity elicited by repeat RNA, and gain of function toxicity elicited by proteins generated from AUG initiated or repeat associated non-AUG initiated (RAN) translation [2, 3].

Fragile X-associated Tremor/Ataxia Syndrome (FXTAS) is a neurodegenerative disease caused by a trinucleotide CGG repeat expansion in the 5′ untranslated region (UTR) of the *FMR1* gene [4]. Clinically, FXTAS is characterized by intention tremor, ataxia, gait abnormalities and cognitive decline [5]. Both patients and CGG knock-in (KI) mouse models of disease have elevated *FMR1* mRNA but lower basal expression of the protein product, FMRP [6, 7]. The pathologic hallmark of FXTAS is the accumulation of ubiquitinated neuronal intranuclear inclusions (NIIs) throughout the brain [8, 9]. NIIs are most prominent in the hippocampus and, to a lesser degree, in the frontal cortex and granule cell layer of the cerebellum [10]. Astrocytic inclusions also occur frequently within the brainstem and other brain regions [8, 10]. Despite their clear role in the clinical syndrome and evidence of cerebellar atrophy on both pathological analysis and imaging, ubiquitinated inclusions are relatively rare in cerebellar Purkinje neurons [11].

In initial work in FXTAS, no single dominant protein species was found in these aggregates [12]. Proteins identified by mass spectrometry and immunohistochemically include, but are not limited to ubiquitinated proteins, lamin A/C, αΒ crystallin, a series of histone proteins and proteasomal subunits, and the RNA binding proteins Sam68, Muscleblind1, and hnRNPA2/B1 [12–15]. In addition, biotinylated antisense RNA probes targeting the 5′ UTR, coding region and 3’UTRs of *FMR1* diffusely stained inclusions in nuclei isolated from FXTAS patient cortex [16]. Based on these initial findings, it was proposed that CGG repeat RNA serves as a nidus for inclusion formation by binding to and sequestering specific proteins into these aggregates. Consistent with this model, many of the factors identified within inclusions to date are RNA binding proteins that associate with CGG repeat RNA in in vitro assays [13–15, 17]. Of note, FMRP itself is not found in the inclusions and loss of the protein is not associated with neurodegeneration in clinical cases or animal models [18, 19].

An alternative mechanism by which inclusions may form in FXTAS is based on a unique form of protein translational initiation known as repeat associated non-AUG (RAN) translation [3, 20]. In rabbit reticulocyte lysates, transfected cells and neurons, *Drosophila*, and mouse models of the disease, expression of the 5′ UTR of *FMR1* leads to RAN translation of a series of homopolymeric proteins, with different efficiencies of production and accumulation in different reading frames [21–26]. RAN translation can occur from both sense strand CGG repeat (producing polyglycine (FMRpolyG), polyalanine (FMRpolyA) and polyarginine (FMRpolyR) repeat containing proteins) and antisense strand CCG repeat (producing polyproline (ASFMRpolyP), polyalanine (ASFMRpolyA), and polyarginine (ASFMRpolyR) containing proteins) mRNA transcripts in reporter assays [23, 27]. FMRpolyG production in particular appears critical for NII formation, as mutations that largely preclude FMRpolyG production in the sequence just 5′ to the repeat prevents NII formation in *Drosophila,* and both CGG KI mice and repeat expressing transgenic mice [22, 24–26]. Moreover, generation of FMRpolyG absent the CGG repeat through use of alternative codons is sufficient to elicit inclusion formation in transfected cells [28]. Importantly, the ability to generate FMRpolyG is predictive of whether CGG repeats expressed in neurons, *Drosophila,* or in transgenic mice are capable of eliciting neurodegeneration despite comparable levels of expression of the repeat containing mRNA [25, 26].

Previous studies have demonstrated the presence of FMRpolyG positive aggregates in FXTAS patient tissue using different mouse monoclonal antibodies targeted against either the N-terminal region or C-terminal region of FMRpolyG [25, 26, 29, 30]. In addition, ASFMRpolyP and ASFMRpolyA staining is detected in some aggregates in FXTAS cases [27]. However, very little FMRpolyA positive staining has been seen previously [25]. The relative abundance of each RAN peptide in FXTAS and their overlap with other pathological markers of inclusion burden has not been systematically evaluated to date.

Here we describe a series of new antibodies generated against FMRpolyG and FMRpolyA epitopes that contain a portion of the homopolymeric repeat. After thoroughly establishing the specificity of these antibodies for their pathological targets, we use them to determine the relative distribution and abundance of different RAN derived proteins in the brains of FXTAS patients and their overlap with ubiquitin and p62 pathology in using blinded analysis to provide unbiased and rigorous results. These studies suggest that FMRpolyG, and not FMRpolyA, is a near-obligate component of NIIs in FXTAS and further support a role for FMRpolyG accumulation in the FXTAS pathology.

## Materials and methods

### FMRpolyG and FMRpolyA polyclonal antibody generation

Rabbit polyclonal antibodies were generated by Abclonal (Cambridge, MA). FMRpolyG peptides were generated corresponded to either the N-terminal or C-terminal region of the predicted protein and extended into the repeat region. The peptide for FMRpolyA also including a portion of the repeat and the C-terminal end of the predicted protein. Exact sequences can be found in Fig. 2 and Fig. 7, respectively. Antibodies were affinity purified from anti-sera. Pre-bleed sera and the peptide were used to characterize the antibodies. Protein concentrations of the pre-bleed sera were determined. The dilution of pre-bleed sera used had an equal protein concentration as the dilution of antibody used. A 100x excess of peptide was incubated with the antibody prior to use to block the antibodies’ ability to bind to antigen.

### Western blotting

HEK293 cells were transfected with constructs expressing FMRpolyG-GFP, ATG-FMRpolyG-GFP, FMRpolyA, or EGFP-N1 (Clontech) using Fugene HD (Promega) following the manufacturer’s protocol [24, 26]. Lysates were collected 24 h after transfection, as previously described [25, 26, 30]. Proteins were separated using SDS-PAGE and transferred to PVDF membrane. Proteins were detected with antibodies against GFP (Sigma, 1:1000) and FMR polyclonal antibodies (11000). GAPDH or tubulin was used as a loading control.

### Immunocytochemistry

HEK293 cells were grown on a 4-well chamber slide and transfected with FMRpolyG-GFP, ATG-FMRpolyG-GFP, FMRpolyA-GFP, or EGFP-N1 using Fugene HD [24, 26]. Cells were fixed with 4% paraformaldehyde 24 h after transfection and permeabilized with 0.1% Triton X-100 in phosphate buffered saline, 10 mM MgCl_2_, 1 mM CaCl_2_ (PBS-MC). 5% normal goat serum in PBS-MC (0.1% Triton) was used as a blocking agent and diluent for primary antibodies. N-terminal FMRpolyG (NTF1, 1:200), C-terminal FMRpolyG, (CTF1, 1:100), and FMRpolyA (1:100) were incubated with anti-GFP (1:1000) overnight at 4 °C. AlexaFluor488 goat anti-mouse and AlexaFluor555 goat anti-rabbit IgG secondary antibodies were used. Nuclei were stained and coverslips mounted using Prolong Gold with DAPI (ThermoFisher). Images were captured on an inverted Olympus (Tokyo, Japan) IX71 microscope at the same exposure and processed using SlideBook 5.5 software.

### Autopsy tissue sources

Control, C9ORF72, SCA3 and FXTAS brain regions were obtained from the University of Michigan Brain Bank, the New York Brain Bank, and the McKnight Brain Institute Brain bank at the University of Florida with informed consent of the patients or their relatives and the approval of the local institutional review boards. FXTAS cases 1–3 were described previously [26, 27]. Case #4 is the subject of a separate manuscript in review (Benoit Giasson, Anthony Yachnis, and Stefan Prokop, personal communication). This case had disease onset at age of 55 and death at age 69 in a male with 96 CGG repeats.

### Immunohistochemistry and co-immunofluorescence

For immunohistochemistry, paraffin embedded sections were de-paraffinized with a series of xylene washes and decreasing concentrations of ethanol. Antigen retrieval (AR) was done with citrate buffer, if necessary. Endogenous peroxidase was quenched with 1% hydrogen peroxide for 30 min. Sections were blocked in 5% normal goat serum (NGS) in Tris, pH 7.6. Primary antibodies, p62 (Proteintech, 1:1000, acid AR), ubiquitin (DAKO, 1:250, basic AR), ubiquilin 2 (Novus Biologicals, 1:200, acid AR), NTF1 (Abclonal, 1:200, no AR), CTF1 (Abclonal, 1:40, basic AR), and FMRpolyA (Abclonal, 1:100, basic AR), 8FM (Sigma, 1:50), 9FM (Sigma, 1:50), were diluted in 5% NGS Tris B (Tris pH 7.6, 0.1% Triton-X 100, 0.5% bovine serum albumin) and were incubated with sections overnight at 4 °C. The following day, sections were washed in Tris A (Tris pH 7.6, 0.1% Triton) and Tris B. Antibody detection was determined using VECTASTAIN Elite ABC HRP kit (Vector Laboratories) following the manufacturer’s protocol. Sections were counterstained with hematoxylin (Vector Laboratories). After dehydrating the slides through a series of increasing concentration of ethanol and then xylenes, coverslips were mounted to the slides using DPX mounting media. Images were taken on an Olympus BX51 microscope.

For co-immunofluorescence, sections were blocked with 5% NGS in PBS-MC. Ubiquitin, ubiquilin 2, p62, NTF1, CTF1, and FMRpolyA were diluted in 5% NGS-PBS-MC, overnight at 4 °C. Goat anti-rabbit and goat anti-mouse IgG antibodies conjugated to AlexaFluor488 or AlexaFluor635 (ThermoFisher, 1:500) were used. Nuclei were stained and coverslips mounted using Prolong Gold with DAPI (ThermoFisher). Images were taken on an Olympus confocal microscope and compiled and analyzed using ImageJ.

### Analysis of immunohistochemistry

Intensity of staining was graded on a five-point integral scale of 0 (no staining) to 3 (intense staining with an additional point added if an aggregate/inclusion was present. Grading was done by three separate reviewers blinded to the genotype of the tissue as well as the identity of the antibody being used. All reviewers were trained and tested to ensure consistent grading for intensities and inclusion identification. A subset of images was repeated among reviewers to assess variability during retest. All images were randomized and anonymized prior to assigning them to the reviewers. An average of 20 images per slide was counted for each of the control and FXTAS cases and multiple brain regions were analyzed. As the presence of more than one inclusion per cell was not observed**,** the number of cells with inclusions were presented as a ratio to the total number of cells of that class in the images evaluated.

A summary of the histology scoring can be found in Table 1. A plus/minus grading scale was used based on the percentage of cells that had a significant amount of staining. If fewer than 1% of cells had significant staining, this was called negative. Low intensity staining had up to 10% of cell stained, moderate intensity up to 20%, high intensity up to 30%, and highly intense and frequent up to 30% and were graded +, ++, +++, and ++++, respectively. To determine the relative distribution of inclusions between glia and neurons, the total number of observed inclusions were separated into glial or neuronal using standard morphological criteria [10]. Neurons were identified by their size, their large centrally placed nuclei with dense heterochromatin and nucleoli, and by their abundant cytoplasm. In contrast, astrocytes, which included oligodendroglia, were identified by their smaller, euchromatic irregular, ovoid nuclei without nucleoli or abundant cytoplasm. Exceptions to this strategy were made for granule cells in the cerebellum and near the edges of samples, where endocytic cells might complicate this interpretation.

**Table 1:** Summary of antibody staining in control and FXTAS patient tissue

Brain Region	Case		Age	Sex	UB	p62	UBQLN2	Nterminal FMRpolyG	Cterminal FMRpolyG
Hippocampus	Control	1	59	N/A	++	+	++	+	+
		2	71	M	+	–	+	–	–
		3	39	M	–	+	–	–	–
		4	47	M	–	+	+	–	–
	FXTAS	1	74	M	++++	++++	+++	++++	+
		2	78	M	+++	++	++++	++++	+
		3	80	M	++++	++	+	++	++
		4	69	M	++++	+++	++	++	++
Cortex	Control	2	71	M	+	–	–	–	–
		4	47	M	+	+	+	+	–
		5	84	M	++++	–	–	+++	–
		6	78	M	++++	–	–	++++	–
	FXTAS	1	74	M	++++	++	+	++++	+
		2	78	M	++	++	–	++++	+
		3	80	M	++++	+	+	+	–

-, negative; +, low-intensity staining; ++, moderate-intensity staining; +++, high-intensity staining; ++++, high-intensity and frequent staining. *UBQLN2* ubiquilin 2, *N/A* not available; M, male

### Statistical analysis

Tabulated scores of the percentage of cells containing an aggregate as well as the staining intensity score were analyzed using a Mann-Whitney U two-tailed t-test with Bonferroni correction for multiple comparisons where appropriate. The Co-localization of immunofluorescence signal within inclusions was analyzed using a Chi-Squared test with Yates correction. All statistical analyses were conducted using GraphPad Prism software.

## Results

**Fig. 1:**
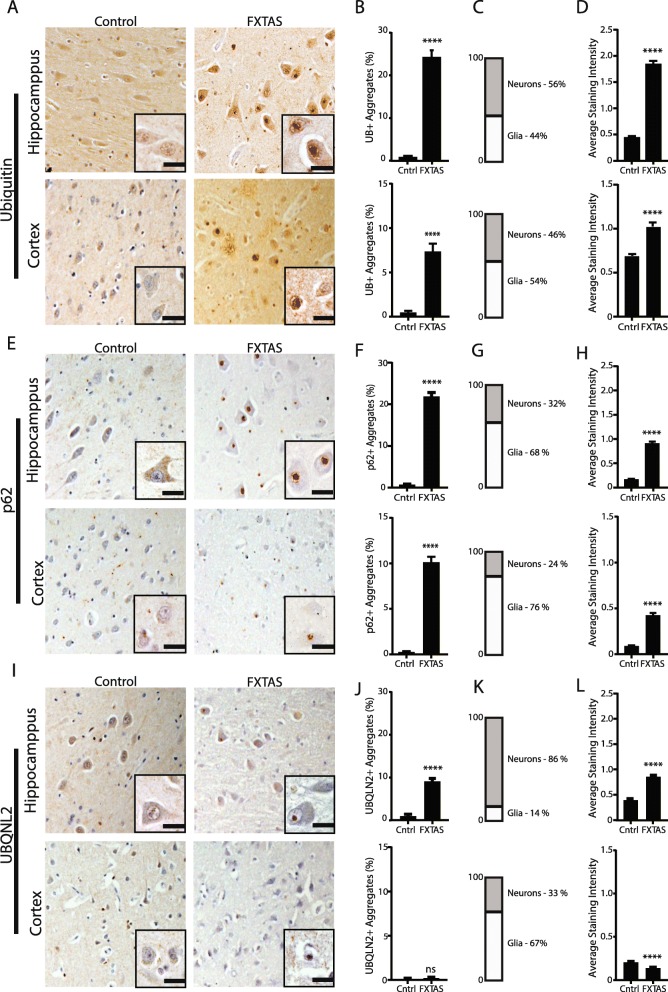
Staining for pathological markers Ubiquitin, p62, and UBQLN2 in FXTAS brain. **a** Representative images from control (left) and FXTAS (right) hippocampus (upper panels) and cortex (lower panels) stained for ubiquitin. Nuclei stained with hematoxylin. Inset- 60x magnification. Scale bar = 20 μm. **b** Quantification of **a** represented as percent neurons with aggregates. Data from hippocampus (top) and cortex (bottom). Results expressed as means ± SEM; Mann-Whitney U-test **** *p* < 0.0001. **c** Graph showing the percentage of cells with ubiquitin positive aggregates that are neurons and glia in hippocampus (top) and cortex (bottom) in FXTAS tissue. **d** Graph comparing the average staining intensity for ubiquitin in hippocampus (top) and cortex (bottom) between control and FXTAS tissue. Results expressed as means ± SEM; Mann-Whitney U-test **** *p* < 0.0001. **e** Representative images from control (left) and FXTAS (right) hippocampus (upper panels) and cortex (lower panels) stained for p62. Nuclei stained with hematoxylin. Inset- 60x magnification. Scale bar = 20 μm. **f** Quantification of **e** represented as percent neurons with aggregates. Data from hippocampus (top) and cortex (bottom). Results expressed as means ± SEM; Mann-Whitney U-test **** *p* < 0.0001. **g** Graph showing the percentage of cells with p62 positive aggregates that are neurons and glia in hippocampus (top) and cortex (bottom) in FXTAS tissue. **h** Graph comparing average staining intensity for p62 in hippocampus (top) and cortex (bottom) between control and FXTAS tissue. Results expressed as means ± SEM; Mann-Whitney U-test **** *p* < 0.0001. **i** Representative images from control (left) and FXTAS (right) hippocampus (upper panels) and cortex (lower panels) stained for UBQLN2. Nuclei stained with hematoxylin. Inset-60x. Scale bar = 20 μm. **j** Quantification of **i** represented as percent of neurons with aggregates. Data from hippocampus (top) and cortex (bottom). Results expressed as means ± SEM; Mann-Whitney U-test **** *p* < 0.0001, ns = not significant. **k** Graph showing the percentage of cells with UBQLN2 positive aggregates that are neurons or glia in hippocampus (top) and cortex (bottom) from FXTAS tissue. **l** Graph comparing average staining intensity for UBQLN2 in hippocampus (top) and cortex (bottom) between control and FXTAS tissue. Results expressed as means ± SEM; Mann-Whitney U-test **** *p* < 0.0001

Ubiquitin, p62/SQSTM1, and ubiquilin 2 (UBQLN2) are components of intracellular inclusions in a number of neurodegenerative diseases, including Huntington disease and C9ORF72-associated ALS/FTD. In non-disease brain, these proteins are generally diffuse within neurons when visualized by immunohistochemistry in both the hippocampus and cortex (Fig. 1a, e and i). Consistent with the original pathological description of FXTAS, we observed significant ubiquitin pathology in FXTAS cases compared to controls (Fig. 1b-d), with intranuclear inclusions observed throughout the hippocampus and cortex, but with relatively fewer inclusions in the brainstem and cerebellum. Aggregates in FXTAS also stained positive for p62 (Fig. 1e-g), consistent with a recent report [31]. We also observed UBQLN2 positive inclusions in FXTAS neuronal nuclei (Fig. 1i-k). Inclusions outside of the nucleus within the neuropil were very rare for all three markers of aggregation.

To quantify the relative accumulation and intensity of staining for these three proteins in FXTAS, we performed quantification of staining for each marker in both FXTAS and control samples from the hippocampus and cortex. Quantification was done by raters who were blinded to the genotype of the case being stained to enhance the rigor of the analysis. A large fraction of FXTAS hippocampal neurons contained inclusions, with ubiquitin, p62, or UBQLN2 aggregates present in 25, 20%, or 8%, of neurons (> 300) counted in each of 4 FXTAS cases, respectively (Fig. 1b, f, and j). This was significantly higher than controls. Inclusions were present in both glia and neurons for all three proteins (Fig. 1c, g, k). Generally, a higher percentage of control neurons have no staining with any of these antibodies. The average staining intensity for each antibody was significantly higher in FXTAS tissue compared to control tissue (Fig. 1d, h, l). Results are summarized in Table 1.

**Fig. 2:**
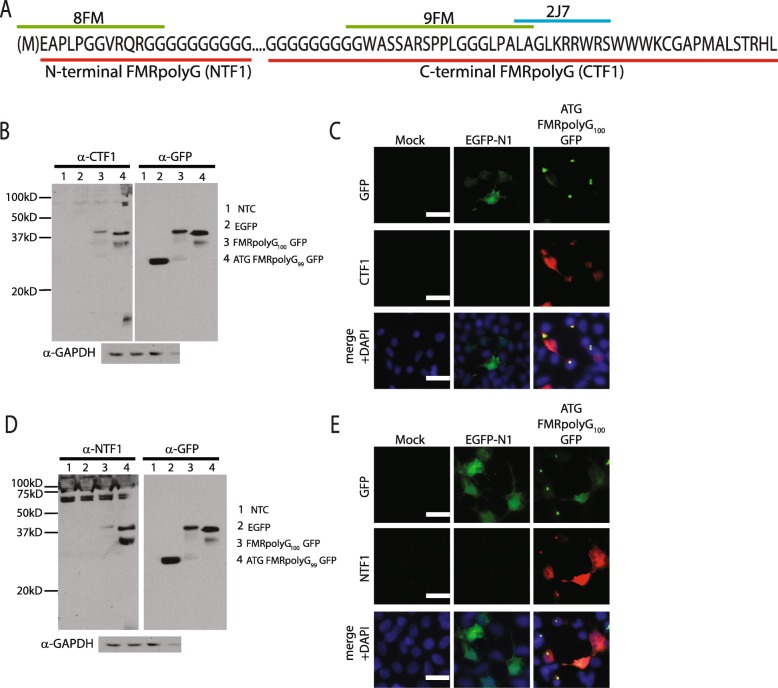
Generation and characterization of new FMRpolyG antibodies. **a** Schematic showing epitopes for published (8FM, 9FM and 2 J7) and novel (NTF1, N-terminal FMRpolyG and CTF1, C-terminal FMRpolyG) antibodies against FMRpolyG. Epitope sequence is underlined (green line - 8FM and 9FM, blue line- 2 J7, red line - NTF1 and CTF1 FMRpolyG antibodies used in this paper). **b** Western blot using CTF1 and GFP antibodies in HEK293 cells transfected with a non-template control (NTC- lanes 1), EGFP-N1 plasmid (lanes 2), FMRpolyG_100_GFP (lanes 3) and ATG FMRpolyG_99_GFP (lanes 4). GAPDH used as loading control. **c** Immunocytochemistry of Mock, EGFP-N1 and ATG FMRpolyG_100_GFP transfected HEK293 cells using CTF1 antibody. Nuclei stained using DAPI. Scale in C = 10 μm. **d** Western blot using NTF1 and GFP antibodies in HEK293 cells transfected with a non-template control (NTC- lanes1), EGFP-N1 plasmid (lanes 2), FMRpolyG_100_GFP (lanes 3) and ATG FMRpolyG_99_GFP (lanes 4). GAPDH used as loading control. **e** Immunocytochemistry of Mock, EGFP-N1 and ATG FMRpolyG_100_GFP transfected HEK293 cells using NTF1 antibody. Nuclei stained using DAPI. Scale in E = 10 μm

To better characterize the role of RAN translation products in FXTAS, we generated a series of new polyclonal antibodies against FMRpolyG and FMRpolyA. Three previously published mouse monoclonal FMRpolyG antibodies were generated using epitopes just N-terminal (8FM) or C-terminal (9FM and 2 J7) to the repeat element. Qualitative analysis in 2 FXTAS cases with 2 J7 estimated that ~ 30% of the inclusions in FXTAS stained positive for FMRpolyG [26]. However, it was unclear whether the remaining inclusions were the result of alternative RAN translation proteins, repeat RNA triggered aggregates, or epitope masking that precluded recognition by the FMRpolyG antibody. Consistent with the latter concept, the N-terminal antibody had greater staining in FXTAS cases [2]. We therefore used larger epitopes involving the N-terminal region (NTF1) and C-terminal region (CTF1) of FMRpolyG, under the assumption that some parts of the protein might be subject to proteolytic cleavage. The exact epitopes used are shown in Fig. 2a, underlined in red. A unique feature of the NTF1 and CTF1 was that each epitope also included a portion of the polyglycine repeat. Western blot analysis of HEK293 cell lysates expressing either EGFP-N1 or FMRpolyG fused to GFP showed both CTF1 and NTF1 antibodies specifically recognized FMRpolyG (Fig. 2b, d). NTF1 had some nonspecific staining compared to CTF1 by immunoblot. By immunofluorescence, both antibodies recognized cells expressing FMRpolyG (Fig. 2c, e).

**Fig. 3:**
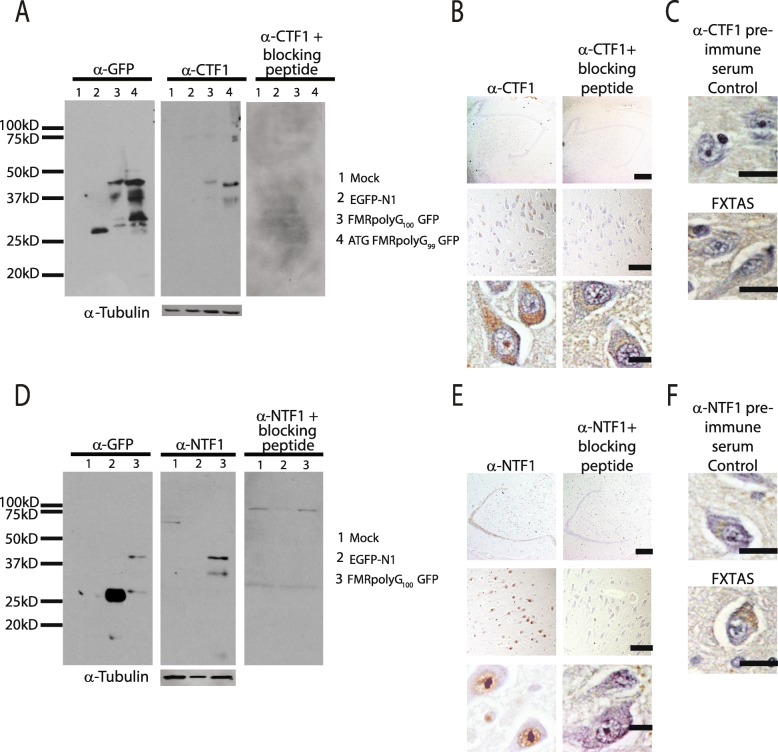
Validation of new FMRpolyG antibodies on FXTAS brain tissue. **a** Western blot using GFP and CTF1 with and without blocking peptide in HEK293 cells that were either mock transfected (lanes1) or transfected with EGFP-N1 plasmid (lanes 2), FMRpolyG _100_GFP (lanes 3) and ATG FMRpolyG_99_GFP (lanes 4). Tubulin used as loading control. **b** Representative brain images from FXTAS patients stained using CTF1 antibody with and without blocking peptide at 4x (top), 20x (middle) and 60x (bottom) magnification. Nuclei stained with hematoxylin. Scale bars are 500 μm, 100 μm and 20 μm respectively. **c** Staining of control and FXTAS brain tissue using pre-bleed serum from CTF1 antibody production. Nuclei stained with hematoxylin. Scale bar = 20 μm. **d** Western blot using GFP and NTF1 with and without blocking peptide in HEK293 cells that were either mock transfected (lanes1) or transfected with EGFP-N1 plasmid (lanes 2) and FMRpolyG_100_GFP (lanes 3). Tubulin used as loading control. **e** Representative brain images from FXTAS patients stained using NTF1 antibody with and without blocking peptide at 4x (top), 20x (middle) and 60x (bottom) magnification. Nuclei stained with hematoxylin. Scale bars are 500 μm, 100 μm and 20 μm respectively. **f** Staining of control and FXTAS brain tissue using pre-bleed serum from NTF1 antibody production. Nuclei stained with hematoxylin. Scale bar = 20 μm

To validate the specificity of these antibodies, we performed a number of additional controls. A potential problem with polyclonal antibodies, in particular, is non-specific binding of the antibody to proteins not containing the antigen. The specificity can be determined by incubating the antibody with a peptide bearing the recognition sequence of the antibody. The antibody would bind the peptide and would not be able to bind the antigen in either protein lysates or human tissue. After incubating both NTF1 and CTF1 with their respective peptides, neither antibody recognized either FMRpolyG containing lysates (Fig. 3a, d) or FXTAS tissue (Fig. 3b, e). Pre-immune sera for CTF1 had no staining in control or FXTAS hippocampal tissue (Fig. 3c) while pre-immune sera for NTF1 had light cytoplasmic staining in both control and FXTAS tissue (Fig. 3f).

**Fig. 4:**
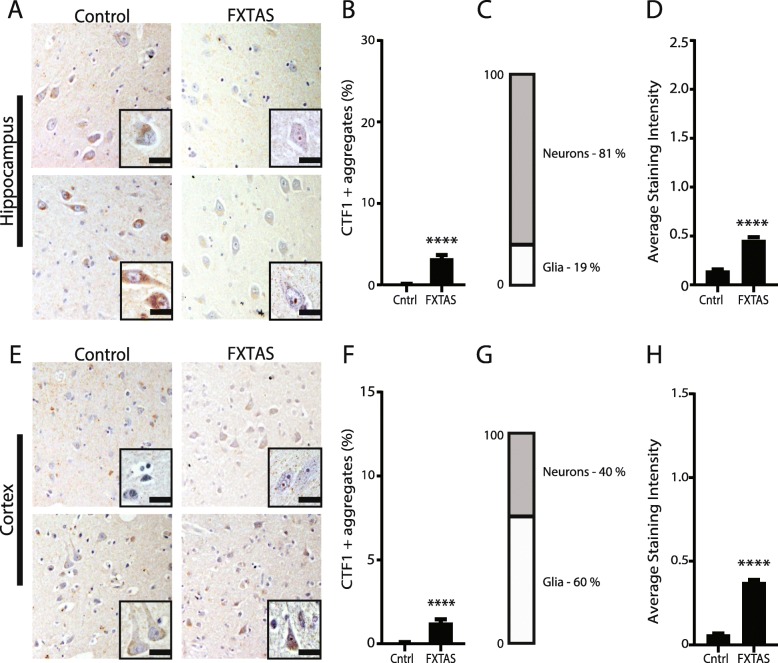
C-terminal targeted FMRpolyG antibody staining in FXTAS tissue. **a** Representative images from control (left) and FXTAS (right) hippocampus stained with CTF1 antibody. Nuclei stained with hematoxylin. Inset- 60x magnification. Scale bar = 20 μm. **b** Quantification of **a** represented as percent neurons with CTF1 positive aggregates in hippocampus. Results expressed as means ± SEM; Mann-Whitney U-test **** *p* < 0.0001. **c** Graph showing the percentage of cells with CTF1 positive aggregates that are neurons or glia in FXTAS hippocampus. **d** Graph comparing average staining intensity for CTF1 positive aggregates between control and FXTAS hippocampus. Results expressed as means ± SEM; Mann-Whitney U-test **** *p* < 0.0001. **e** Representative images from control (left) and FXTAS (right) cortex stained for CTF1 positive aggregates. Nuclei stained with hematoxylin. Inset- 60x magnification. Scale bar = 20 μm. **f** Quantification of **e** represented as percent neurons with aggregates in cortex. Results expressed as means ± SEM; Mann-Whitney U-test **** *p* < 0.0001. **g** Graph showing the percentage of cells with CTF1 positive aggregates that are neurons or glia in FXTAS cortex. **h** Graph comparing average staining intensity for CTF1 positive aggregates between control and FXTAS cortex. Results expressed as means ± SEM; Mann-Whitney U-test **** *p* < 0.0001

With the newly validated antibodies, we looked to see if they could recognize FMRpolyG in patient FXTAS brain tissue. In hippocampal and cortical neurons, CTF1 exhibited substantial cytoplasmic staining in both control and FXTAS tissue (Fig. 4a, e). CTF1 positive inclusions were relatively rare, with approximately 3 and 1.2% of FXTAS neurons exhibiting CTF1 positive aggregates in the hippocampus and cortex, respectively, by blinded quantification (Fig. 4b, f). This was significantly greater than that observed in control tissues. The overall intensity of staining was significantly higher in FXTAS cortex and hippocampus compared to control (Fig. 4d, h). Most CTF positive inclusions were in neurons (Fig. 4c, g).

**Fig. 5:**
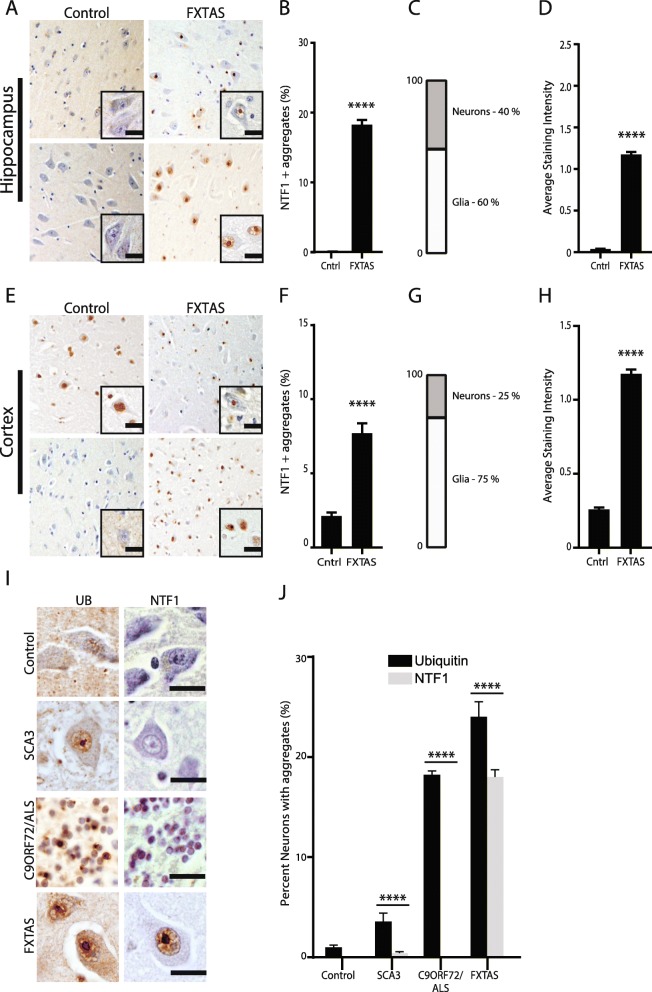
N-terminal targeted FMRpolyG antibody staining in FXTAS tissue. **a** Representative images from control (left) and FXTAS (right) hippocampus stained with NTF1 antibody. Nuclei stained with hematoxylin. Inset- 60x magnification. Scale bar = 20 μm. **b** Quantification of **a** represented as percent neurons with NTF1 positive aggregates in hippocampus. Results expressed as means ± SEM; Mann-Whitney U-test **** *p* < 0.0001. **c** Graph showing the percentage of cells with NTF1 positive aggregates that are neurons or glia in FXTAS hippocampus. **d** Graph comparing average staining intensity for NTF1 positive aggregates between control and FXTAS hippocampus. Results expressed as means ± SEM; Mann-Whitney U-test **** *p* < 0.0001. **e** Representative images from control (left) and FXTAS (right) cortex stained with NTF1 antibody. Nuclei stained with hematoxylin. Inset- 60x magnification. Scale bar = 20 μm. **f** Quantification of E represented as percent neurons with NTF1 positive aggregates in cortex. Results expressed as means ± SEM; Mann-Whitney U-test **** *p* < 0.0001. **g** Graph showing the percentage of cells with NTF1 positive aggregates that are neurons or glia in FXTAS cortex. **h** Graph comparing average staining intensity for NTF1 positive aggregates between control and FXTAS cortex. Results expressed as means ± SEM; Mann-Whitney U-test **** *p* < 0.0001. I Representative images showing UB & NTF1 staining of control, SCA3, C9ORF72/ALS-FTD and FXTAS brain. Nuclei stained with hematoxylin. Scale bars = 20 μm. **j** Quantification of **i** showing percent neurons with UB and NTF1 positive aggregates in control, FXTAS, C9ORF72/ALS-FTD and SCA3 tissues. Results expressed as means ± SEM; Mann-Whitney U-test **** *p* < 0.0001

In contrast to the CTF1 antibody, NTF1 staining of FMRpolyG was qualitatively different. Staining of hippocampal and cortical neurons with NTF1 showed low levels of cytoplasmic staining in control tissue. However, in some control cases, cortical neurons had moderate diffuse nuclear staining without aggregate formation. In FXTAS cases, NTF1 avidly stained intranuclear FMRpolyG positive aggregates in both the hippocampus and cortex (Fig. 5a, e). The percentage of neurons with FMRpolyG NTF1 positive aggregates (17%) was similar to the percentages seen with ubiquitin (25%) and p62 (21%) and was significantly higher than control tissue (Fig. 5b). In the cortex, fewer total neurons had aggregates but the percentage of neurons with FMRpolyG aggregates in FXTAS cases was comparable to staining with Ubiquitin and p62 and much greater than control cases (Fig. 5f). Quantification of the average staining intensity in FXTAS cortex and hippocampus was significantly higher than in controls by analysis performed blinded to genotype and antibody evaluated (Fig. 5d, h). A majority of NTF1 positive inclusions were found in glia in the cortex and hippocampus (Fig. 5c, g).

To confirm the specificity of NTF1 for FMRpolyG, this antibody was also used to stain tissue from the pons of one SCA3 patient and from the cerebellum of two C9ORF72 repeat expansion cases who had ALS/FTD. The pons is a region of the brain known to have ubiquitinated aggregates in SCA3 [32]. Moreover, dipeptide repeat proteins accumulate in large ubiquitin and p62 positive inclusions in the cerebellum of most cases with C9ORF72 repeat expansions [33, 34]. When this region was stained with NTF1, nuclear aggregates were not observed in SCA3 patient tissue or C9ORF2 tissue, despite the detection of such inclusions by anti-ubiquitin antibodies (Fig. 5i, j).

**Fig. 6:**
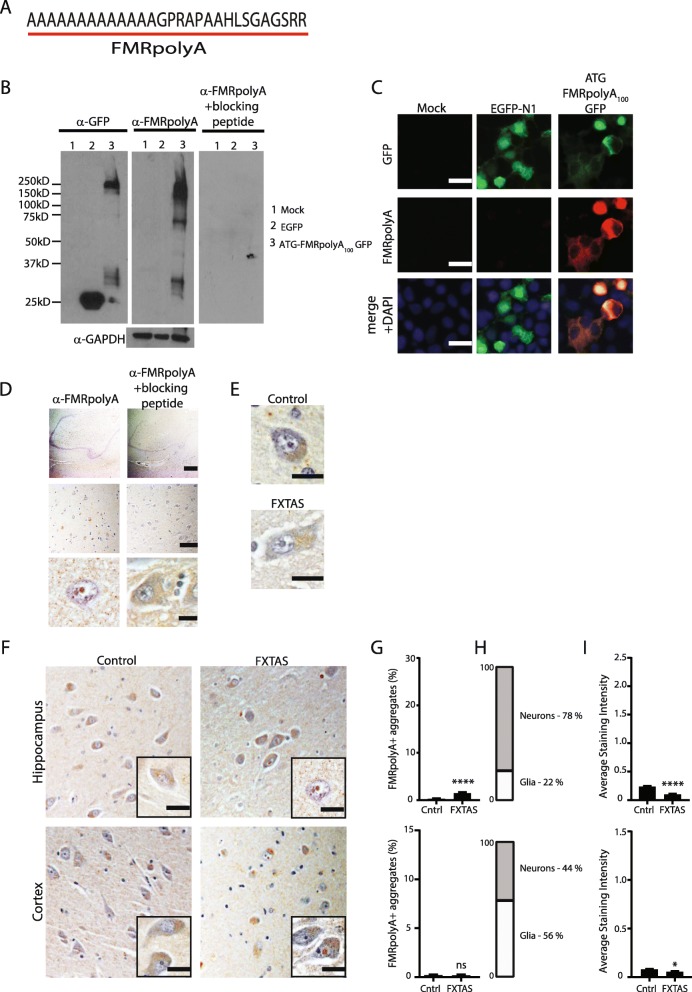
FMRpolyA staining in control and FXTAS tissues. **a** Protein sequence of FMRpolyA epitope. **b** Western blot using FMRpolyA antibody with and without blocking peptide and GFP in HEK293 cells that were either mock transfected (lanes1), transfected with EGFP-N1 plasmid (lanes 2) and ATG FMRpolyA_100_ GFP (lanes 3). GAPDH used as a loading control. **c** Immunocytochemistry of Mock, EGFP-N1 and FMRpolyA_100_GFP transfected HEK cells using FMRpolyA antibody. Nuclei stained using DAPI. Scale bar is = 10 μm. **d** Representative brain images from FXTAS patients stained using FMRpolyA antibody with and without blocking peptide at 4x (top), 20x (middle) and 60x (bottom) magnification. Nuclei stained with hematoxylin. Scale bars are 500 μm, 100 μm and 20 μm respectively. **e** Staining of control and FXTAS brain tissue using pre-bleed serum from FMRpolyA antibody production. Nuclei stained with hematoxylin. Scale bar = 20 μm. **f** Representative images from control (left) and FXTAS (right) hippocampus (upper panels) and cortex (lower panels) stained with FMRpolyA antibody. Nuclei stained with hematoxylin. Inset- 60x magnification. Scale bar = 20 μm. **g** Quantification of F represented as percent neurons with aggregates. Data from hippocampus (top) and cortex (bottom). Results expressed as means ± SEM; Mann-Whitney U-test **** *p* < 0.0001. **h** Graph showing the percentage of cells with FMRpolyA positive aggregates that are neurons or glia in hippocampus (top) and cortex (bottom) from FXTAS tissue. **i** Graph comparing average staining intensity for FMRpolyA between control and FXTAS hippocampus (top) and cortex (bottom). Mann-Whitney U-test * *p* < 0.05, ns = not significant

Another protein generated by RAN translation from reporter constructs is FMRpolyA [23, 24, 26]. As with FMRpolyG, the contribution of FMRpolyA to FXTAS pathology was determined. A polyclonal antibody was generated against the C-terminal fragment of FMRpolyA (Fig. 6a). By western blot and immunocytochemical analysis, the antibody specifically recognized FMRpolyA in transfected HEK293 cells (Fig. 6b, c). Incubating the antibody against FMRpolyA with the corresponding peptide prevented the antibody from recognizing FMRpolyA in HEK293 lysates or in human brain tissue (Fig. 6b, d). The pre-immune serum has some light cytoplasmic staining in control and FXTAS patient tissue but aggregates were not seen (Fig. 6e).

FMRpolyA exhibited light cytoplasmic staining in both control and FXTAS tissue. FMRpolyA positive intranuclear aggregates were rare in hippocampal neurons (1.5%) but were observed significantly more frequently in FXTAS cases compared to controls. In contrast, intranuclear inclusions were not observed at an increased frequency in FXTAS compared to control cortical neurons by rated blinded assessment (Fig. 6f, g). The average staining intensity was actually decreased in FXTAS tissue compared to control tissue (Fig. 6h). These data, along with previous published findings [25], suggest FMRpolyA is not a major component of intranuclear inclusions in FXTAS.

**Fig. 7:**
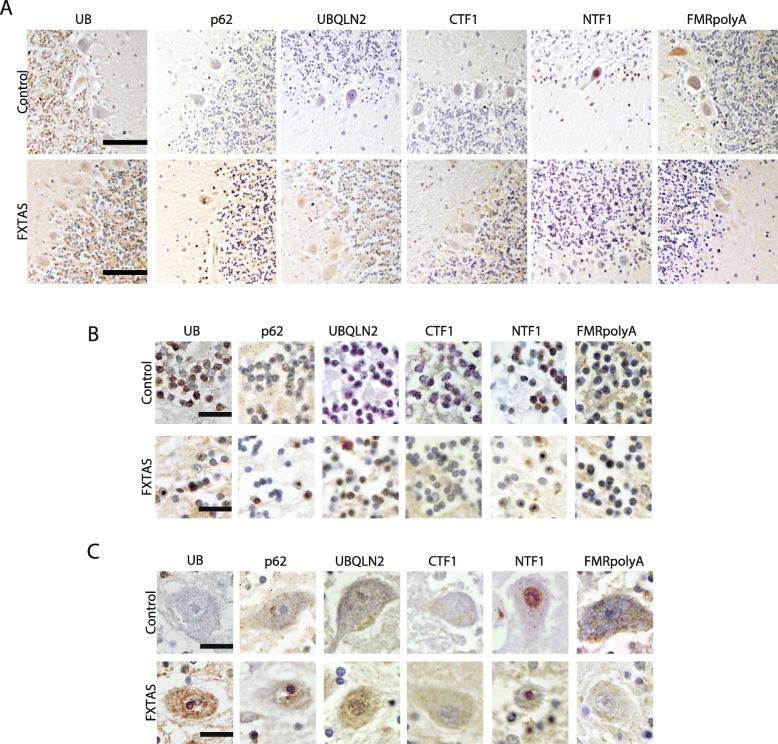
Cerebellar staining for FMRpolyG and other pathological markers. **a** Representative 20x images of cerebellum stained with the indicated antibodies from control and FXTAS brain tissues. Scale bar is 100 μm. **b** Representative 60x images from the granular cell layer of control and FXTAS patients stained with the indicated antibodies. Scale bar = 20 μm. **c** Representative 60x images from the Purkinje cell layer of control and FXTAS patients stained with the indicated antibodies. Scale bar = 20 μm

Neurodegeneration of the cerebellum is a common feature in FXTAS cases. We therefore used these antibodies on patient cerebellar tissue (Fig. 7) In FXTAS, ubiquitin, p62, and NTF1 stained aggregates throughout the granular layer of the cerebellum (Fig. 7b). Rare aggregates were seen in Purkinje cells with ubiquitin and NTF1 (Fig. 7c), but these were less abundant than staining observed in the granule cell layer, a finding largely consistent with published studies.

**Fig. 8:**
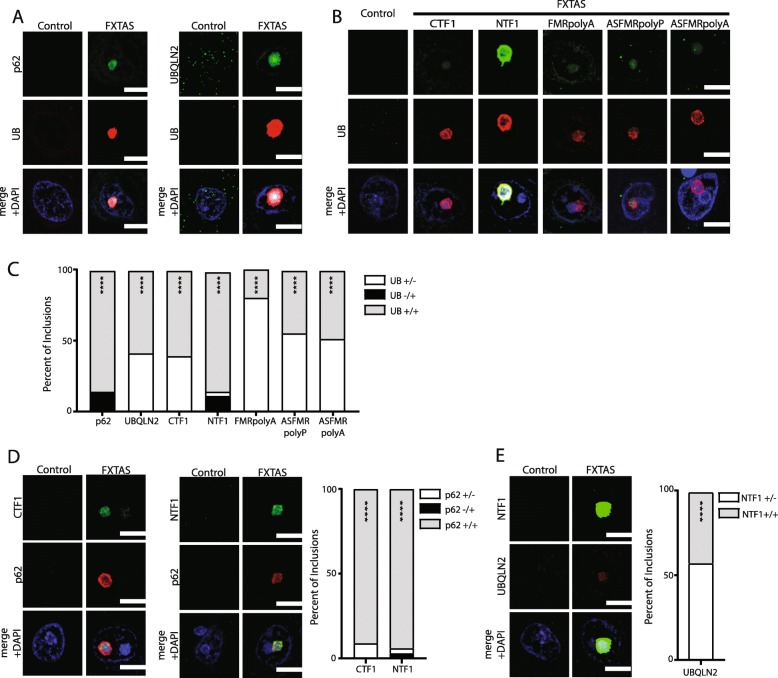
Co-localization of FMR antibodies. **a** Immunofluorescence in control and FXTAS brain tissue co-stained with UB and p62 (left panels) and UB and UBQLN2 (right panels). Nuclei were stained with DAPI. Scale bar = 10 μm. **b** Immunofluorescence in control and FXTAS brain tissue co-stained with UB and either CTF1, NTF1, FMRpolyA, ASFMRpolyP or ASFMRpolyA. Nuclei were stained with DAPI. Scale bar = 10 μm. **c** Graph comparing the percent of inclusions that were either UB positive and co-stain negative, UB negative and co-stain positive, or both UB positive and co-stain positive. Chi-squared test with Bonferroni Correction for multiple comparisons. *N* > 50 inclusions/comparison. **** *p* < 0.0001. **d** Immunofluorescence in control and FXTAS brain tissue co-stained with p62 and CTF1 (left panels) and p62 and NTF1 (middle panels). Nuclei were stained with DAPI. Scale bar = 10 μm. Graph comparing the percent of inclusions that were either p62 positive and CTF1/NTF1 negative, UB negative and CTF1/NTF1 positive, or UB positive and CTF1/NTF1 positive (right). Chi-squared test with Bonferroni correction for multiple comparisons. N > 50cells/comparison. **** *p* < 0.0001. **e** Immunofluorescence in control and FXTAS brain tissue co-stained with NTF1 and UBQLN2. Nuclei were stained with DAPI. Scale bar = 10 μm. Graph comparing the percent of inclusions positive for NTF1 and UBQLN2 (right). Chi-squared test with Bonferroni correction. **** *p* < 0.0001

To see if these antibodies are staining the same aggregates, co-immunofluorescence was performed. NTF1 and CTF1 stain the same aggregates as previously published antibodies 9FM and 8FM, respectively, although the intensity of staining and sensitivity for detection of inclusions were greater for NTF1 in particular (Additional file 1). In FXTAS hippocampal neurons, ubiquitin positive aggregates were also positive for p62, UBQLN2, NTF1, CTF1, FMRpolyA as well as ASFMRpolyP and ASFMRpolyA at ratios greater than expected by chance, even after correction for multiple comparisons (Fig. 8a-c, *n* > 50 inclusions/comparison). Nearly all ubiquitin aggregates also stained positive for p62 and FMRpolyG with NTF1 whereas only 58 and 20% were positive for UBQLN2 or FMRpolyA, respectively (Fig. 8b, c). Intriguingly, 14% of p62+ and 11% of NTF1+ inclusions were negative for ubiquitin (Fig. 8c, black bars). Consistent with this, p62 co-localized to the same aggregates as NTF1 and CTF1 (Fig. 8d). In contrast, only 42% of NTF1 inclusions were UBQLN2 +, although effectively all UBQLN+ inclusions also stained with NTF1 (Fig. 8e).

## Discussion

FXTAS is an under-recognized inherited neurodegenerative condition which lacks effective therapeutic options [4]. Accurately defining the pathology in patients provides insight into how transcribed CGG repeat expansions elicit toxicity and drive neuronal death. Here we have evaluated a series of pathological markers predominantly in the hippocampus, cortex and cerebellum of FXTAS cases compared to controls in a rater-blinded fashion. These studies reveal that p62 and ubiquitin positive inclusions are present in a large percentage of neurons, with the greatest burden observed in pyramidal hippocampal neurons, consistent with past studies [8, 10, 31]. We find that FMRpolyG is detectable in the vast majority of these inclusions and in ~ 20% of all hippocampal neurons overall in FXTAS patients. In contrast, other RAN translation proteins generated from *FMR1* and *ASFMR1* mRNA are detectable in a much lower percent of inclusions, as is the ALS and autophagy-associated protein UBQLN2. In the context of published studies demonstrating that FMRpolyG is the most abundantly translated RAN protein from CGG repeats and that production of FMRpolyG is required for inclusion formation in *Drosophila,* transfected cells and both CGG KI and CGG transgenic mice [22, 25, 26], these data are consistent with a disease model where FMRpolyG production is a central contributor to ubiquitinated neuronal intranuclear inclusion formation in FXTAS.

We used two new polyclonal antibodies to FMRpolyG directed to the N-terminus (NTF1) and the C-terminus (CTF1) of the protein. Intriguingly, these two antibodies did not have the same patterns of staining. Specifically, NTF1 had diffuse nuclear staining in control cortex and hippocampus and robustly stained inclusions throughout the brain. In contrast, CTF1 had mild staining predominantly in the cytoplasm in control cases and stained inclusions in a lower fraction of neurons in FXTAS cases. The cause of this discrepancy is not yet known. Control studies using pre-immune sera or pre-treatment with a blocking peptide demonstrate that staining by both antibodies is specific to their epitopes. Moreover, NTF1 does not stain ubiquitinated inclusions in two different neurodegenerative disease cases, SCA3 and C9ORF72 associated ALS/FTD. In addition, both epitopes are unique within the proteome and fragments of their epitopes do not clearly overlap with other proteins. These differences could reflect different affinities of the antibodies for their epitope targets. Arguing against this hypothesis is the finding that these staining patterns appear consistent with previously published antibodies, where monoclonal FMRpolyG C-terminal targeted antibodies have lower staining of inclusions and a diffuse cytoplasmic pattern in FXTAS tissues while the N-terminal targeted antibody more robustly stained inclusions [17, 26, 29]. Access to the indicated epitopes in tissue may be different due to protein interactions or characteristics of the inclusions formed. Alternatively, FMRpolyG may undergo proteolytic cleavage in cells into smaller fragments that remove the C-terminal component of the protein and leave an N-terminal fragment consisting mostly of the polyglycine expansion fragment. Of note, this polyglycine component is critical for inclusion formation in overexpression systems [25, 26, 28].

When the 5’UTR of *FMR1* is placed upstream of GFP or nanoluciferase and expressed from plasmids or in vitro transcribed mRNA, FMRpolyG production occurs at both normal and expanded CGG repeat sizes in the absence of an AUG codon, suggesting that the protein could be made normally from *FMR1* mRNA [23, 25, 26]. Consistent with this hypothesis, we observe that both NTF1 and CTF1 have some staining in control cortical and hippocampal neurons. Whether FMRpolyG produced from normal sized repeats has a function is unknown. Previous work suggests that FMRpolyG interacts with a variety of proteins, including the nuclear lamin associated factor LAP2β [25]. However, the interactome of this protein was defined in the setting of fusion of FMRpolyG to GFP with an expanded CGG repeat and with overexpression in HEK293 cells. As such, the relevance of these interactors to the native repeat size in human neurons or brain is not clear. Future studies will be needed to define what, if any, functions and interactions FMRpolyG has in neurons under normal contexts.

We also describe a new antibody raised against FMRpolyA. This protein is made less efficiently than FMRpolyG in RAN translation reporter assays and its production is much more dependent on an expanded CGG repeat [23]. Moreover, it is significantly less aggregation prone than FMRpolyG in GFP fusion studies in transfected cells [26]. Consistent with these features, we observe very little FMRpolyA in control or FXTAS brains and only a small percentage of inclusions stain positive for FMRpolyA. The staining observed is equivalent to or less than that observed even for RAN translation products generated from *ASFMR1* [27], whose transcript is produced at a significantly lower abundance that *FMR1* in both patients and controls. These findings are consistent with a prior study suggesting FMRpolyA is not readily observed in most FXTAS inclusions [25]. While these studies do not rule out a role for FMRpolyA in disease pathogenesis, they do suggest that it is not a central nucleator or major component of ubiquitinated inclusions in FXTAS.

This study has some limitations. Due to tissue access issues, we could not evaluate all of the antibodies described across all brain tissues and regions. In addition, the study used a relatively small cohort of cases and controls. As such, the generalizability of the findings to other FXTAS cases and premutation carriers may be limited. In addition, the ideal control tissue for these studies would have been brain samples from a fully methylated Fragile X Syndrome patient, as such a patient would presumably produce no *FMR1* or *ASFMR1* mRNA. However, such cases are rare and were not available to us. As such, a potential component of non-specific staining for FMRpolyG in control human brains in particular cannot be ruled out.

In summary, this study describes three new antibodies raised against epitopes from RAN translation products generated from CGG repeat expansions in *FMR1* mRNA. They confirm that FMRpolyG is a prominent component of ubiquitinated inclusions in FXTAS. These new antibodies will be a valuable resource to the research community and extend previous studies suggesting a role for RAN translation in the pathogenesis of FXTAS and other nucleotide repeat expansion disorders.

## Additional file

Additional file 1 Co-localization of N-terminal and C-terminal FMRpolyG antibodies. A Immunofluorescence in control and FXTAS brain tissue stained with CTF1 and 9FM antibody (left). Nuclei were stained with DAPI. Scale bar = 10 μm. Graph comparing the percent of inclusions positive for CTF1 and 9FM (right). Chi-squared test. **** *p* < 0.0001. B Immunofluorescence in control and FXTAS brain tissue stained with NTF1 and 8FM antibody (left). Nuclei were stained with DAPI. Scale bar = 10 μm. Graph comparing the percent of inclusions positive for NTF1 and 8FM (right). Chi-squared test. **** *p* < 0.0001.

## Abbreviations

AR: Antigen retrieval
CTF1: C-terminal fragment 1 of FMRpolyG
FXTAS: Fragile X-associated Tremor/Ataxia Syndrome
GAPDH: Glyceraldehyde 3-phosphate dehydrogenase
GFP: Green fluorescent protein
KI: Knock-in
NGS: Normal goat serum
NIIs: Neuronal intranuclear inclusions
NTF1: N-terminal fragment 1 of FMRpolyG
PBS-MC: Phosphate buffered saline with magnesium and calcium chloride
PVDF: Polyvinylidene difluoride
RAN: Repeat associate non-AUG translation
SDS-PAGE: Sodium dodecyl sulfate polyacrylamide gel electrophoresis
UTR: Untranslationed region
