# Supplementary figures and images for: Neuropathology of RAN translation proteins in fragile X-associated tremor/ataxia syndrome
Source: Acta Neuropathol Commun. 2019 Oct 30;7:152. doi: 10.1186/s40478-019-0782-7 (PMC6821001; doi:10.1186/s40478-019-0782-7)

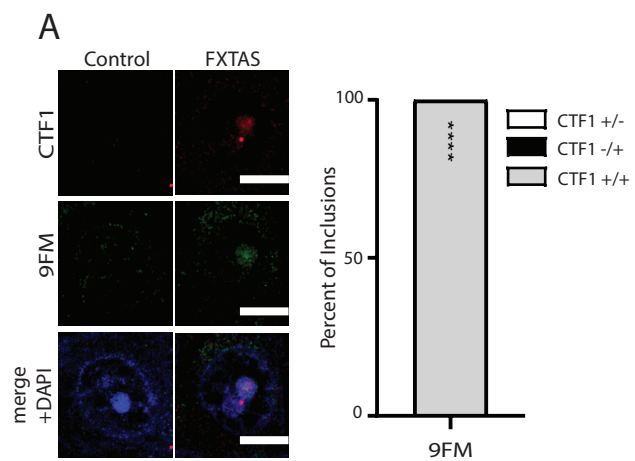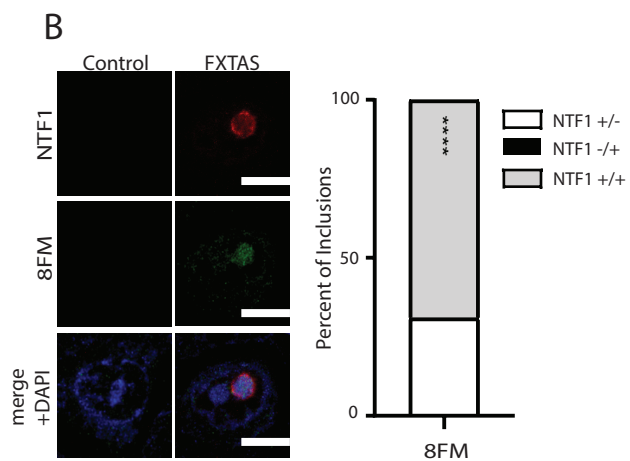

Supplement: Supplementary file 1 — Co-localization of N-terminal and C-terminal FMRpolyG antibodies. A Immunofluorescence in control and FXTAS brain tissue stained with CTF1 and 9FM antibody (left). Nuclei were stained with DAPI. Scale bar = 10 μm. Graph comparing the percent of inclusions positive for CTF1 and 9FM (right). Chi-squared test. **** p < 0.0001. B Immunofluorescence in control and FXTAS brain tissue stained with NTF1 and 8FM antibody (left). Nuclei were stained with DAPI. Scale bar = 10 μm. Graph comparing the percent of inclusions positive for NTF1 and 8FM (right). Chi-squared test. **** p < 0.0001. [file 40478_2019_782_MOESM1_ESM.pdf]
